# REAC technology and hyaluron synthase 2, an interesting network to slow down stem cell senescence

**DOI:** 10.1038/srep28682

**Published:** 2016-06-24

**Authors:** Margherita Maioli, Salvatore Rinaldi, Gianfranco Pigliaru, Sara Santaniello, Valentina Basoli, Alessandro Castagna, Vania Fontani, Carlo Ventura

**Affiliations:** 1 Center for developmental biology and reprogramming - CEDEBIOR, Department of Biomedical Sciences, University of Sassari Viale San Pietro 43/B, 07100 Sassari, Italy; 2 Istituto di Ricerca Genetica e Biomedica, Consiglio Nazionale delle Ricerche (CNR), Monserrato, Cagliari, Italy; 3 Department of Regenerative Medicine, Rinaldi Fontani Institute, Viale Belfiore 43, 50144 Florence, Italy; 4 National Institute of Biostructures and Biosystems at the Department of Experimental, Diagnostic and Specialty Medicine, S. Orsola - Malpighi Hospital, University of Bologna, Via Massarenti 9, 40138 Bologna, Italy; 5 Department of Anti Aging Medicine, Rinaldi Fontani Institute, Viale Belfiore 43, 50144 Florence, Italy; 6 Research Department, Rinaldi Fontani Foundation, Viale Belfiore 43, 50144 Florence, Italy; 7 Department of Biotechnology, University of Natural Resources and Life Sciences Vienna, Muthgasse 18, A-1190 Vienna, Austria; 8 Stem Wave Institute for Tissue Healing (SWITH), Ettore Sansavini Health Science Foundation- NPO, via Provinciale per Cotignola 9, 48022 Lugo (Ravenna), Italy

## Abstract

Hyaluronic acid (HA) plays a fundamental role in cell polarity and hydrodynamic processes, affording significant modulation of proliferation, migration, morphogenesis and senescence, with deep implication in the ability of stem cells to execute their differentiating plans. The Radio Electric Asymmetric Conveyer (REAC) technology is aimed to optimize the ions fluxes at the molecular level in order to optimize the molecular mechanisms driving cellular asymmetry and polarization. Here, we show that treatment with 4-methylumbelliferone (4-MU), a potent repressor of type 2 HA synthase and endogenous HA synthesis, dramatically antagonized the ability of REAC to recover the gene and protein expression of Bmi1, Oct4, Sox2, and Nanog in ADhMSCs that had been made senescent by prolonged culture up to the 30^th^ passage. In senescent ADhMSCs, 4-MU also counteracted the REAC ability to rescue the gene expression of TERT, and the associated resumption of telomerase activity. Hence, the anti-senescence action of REAC is largely dependent upon the availability of endogenous HA synthesis. Endogenous HA and HA-binding proteins with REAC technology create an interesting network that acts on the modulation of cell polarity and intracellular environment. This suggests that REAC technology is effective on an intracellular niche level of stem cell regulation.

Cell polarity is crucial in the physiological modulation of stem cell differentiation and aging, as shown by the fact that altered cell polarization invariantly associates with disease, pathological aging and cancer^1^,^2^,^3^. Hyaluronic acid (HA) plays a fundamental role in cell polarity and hydrodynamic processes, affording significant modulation of proliferation, migration, morphogenesis and senescence^4^,^5^,^6^,^7^,^8^,^9^, with deep implication in the ability of stem cells to execute their differentiating plans^10^,^11^. The Radio Electric Asymmetric Conveyer (REAC) technology is aimed to optimize the ion fluxes at the molecular level in order to optimize the molecular mechanisms driving cellular asymmetry and polarization.

Impairment of differentiation observed in senescent cells is largely dependent upon the lack of growth factor-mediated induction of type 2 hyaluronan synthase (HAS2), with subsequent decrease in HA synthesis^12^. Age-related loss of differentiation has been shown to be significantly restorable by HAS2 overexpression^13^, and inhibition of miRNA-7, which is upregulated in aged cells, was found to restore Ha-mediated membrane motility through a HAS2-dependent mechanism^14^.

HA-mediated signaling is also essential for the regulation of cell polarization which occurs in response to stimuli that promote non-symmetrical subcellular organization to fulfill functional requirements emerging during migration, adhesion, or mitotic spindle orientation^15^,^16^. Within this context, we found that treatment of adipose derived human mesenchymal stem cells (ADhMSCs) with REAC technology, was able to invert human stem cell senescence through the activation of both a telomerase-dependent pathway and a telomerase-independent signaling^17^, rescuing a multilineage differentiating potential^18^. Gaining further insights into the antisenescence effect of REAC may pave the way for future anti-aging approaches in degenerative diseases.

Here, we investigated whether senescence reversal operated by REAC may occur through the activation of HAS2. For this purpose, the effect of REAC technology was assessed in ADhMSCs expanded *in vitro* up to the 30^th^ passage in the absence or presence of 4-methylumbelliferone (4-MU), a powerful inhibitor of HA synthesis acting through HAS2 repression^19^,^20^.

## Results

**Figure 1:**
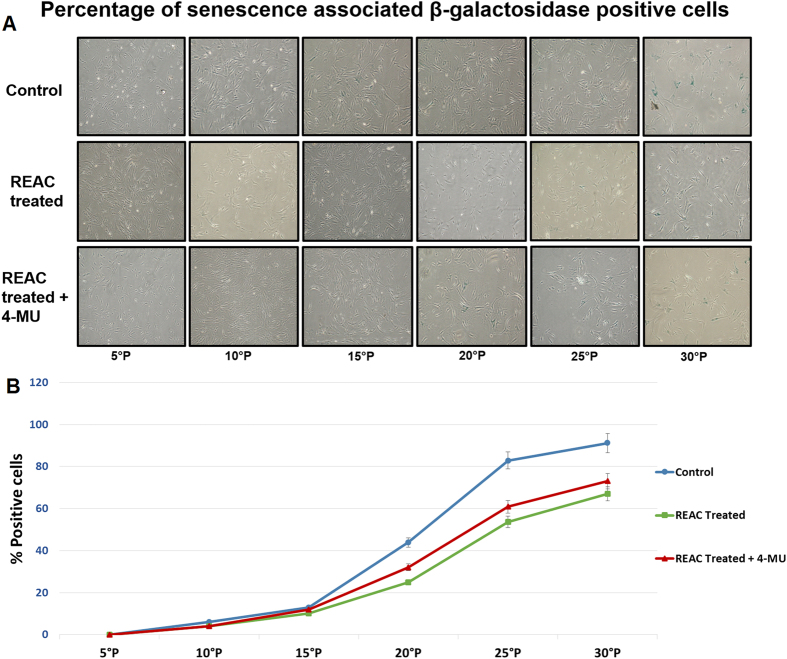
HAS2 inhibition counteracts REAC-mediated decrease in-SA-Gal staining. At the indicated passages, confluent (80%) ADhMSCs were exposed for 12 hours in the absence (Control) or presence of REAC (REAC treated), or they were subjected to a 12-hour REAC treatment in the presence of 1 mM 4-MU (HAS2 inhibitor) (REAC treated + 4-MU). Panel A, shows representative (six separate experiments) SA-β-Gal staining (blue color). Panel B, reports the percentage analysis of positively stained cells under each experimental condition (mean ± S.E.; n = 6; P < 0.05).

Upon prolonged expansion for multiple passages, ADhMSCs underwent replicative senescence, as it was confirmed by the expression of senescence associated β-galactosidase (SA-β-Gal) (Fig. 1). We have previously shown that in ADhMSCs the expression of this marker of senescence is accompanied by a decrease in adipogenic, osteogenic and vascular differentiation^18^. Interestingly, REAC treatment induced a significant decrease in the number of blue-stained senescent cells, as compared to control untreated cells, particularly evident after passage 15^th^ (Fig. 1). When cells were treated with REAC in the presence of 4-MU the number of blue stained ADhMSCs was substantially superimposable to the number of SA-β-Gal-positive cells observed in REAC-untreated cells (Fig. 1).

**Figure 2:**
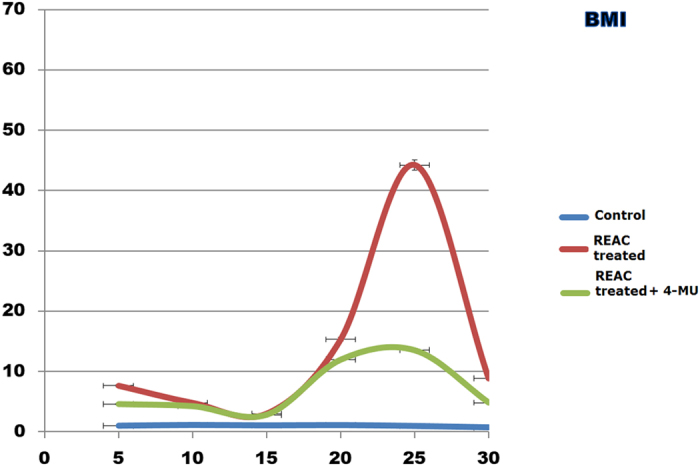
HAS2 inhibition antagonizes REAC-mediated action on Bmi1 gene expression. At each indicated passage, ADhMSCs were left untreated (control) or treated for 12 hours with REAC, with or without 1 mM 4-MU. The mRNA level of Bmi1 was normalized to HPRT1 and was expressed as fold change relative to mRNA level at time 0 (untreated cells at passage 5), defined as 1. At each time point, mRNA levels from REAC-treated ADhMSCs were significantly different from those detected in control untreated cells. mRNA levels from cells that had been treated with REAC in the presence of 4-MU were significantly different from the expression levels detected in REAC-treated cells in the absence of the HAS2 inhibitor. (mean ± S.E.; n = 6; P < 0.05).

**Figure 3:**
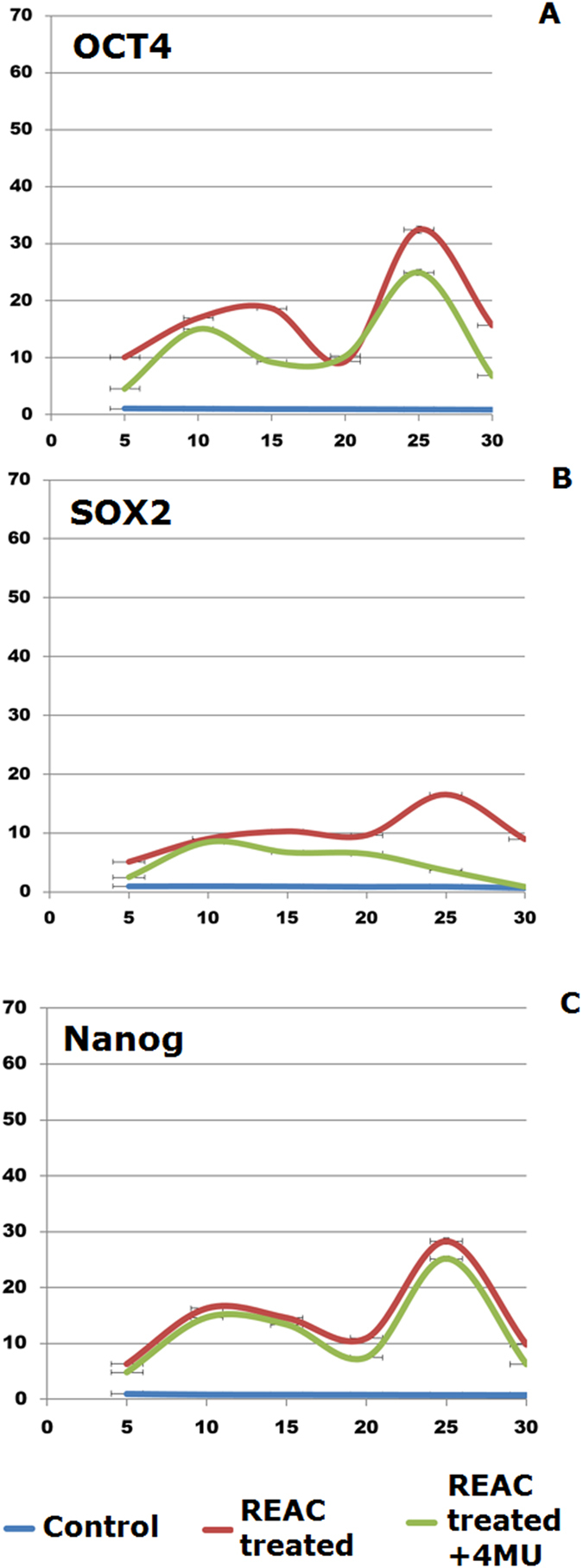
HAS2 inhibition blunts the effect of REAC treatment on the transcription of stemness related genes. At each indicated passage, ADhMSCs were left untreated (control) or subjected to REAC treatment for 12 hours, in the absence or presence of 1 mM 4-MU. The mRNA levels of Oct4 (**A**), Sox2 (**B**), or Nanog (**C**) were normalized to HPRT1 and were expressed as fold of change relative to mRNA level at time 0 (unexposed cells at passage 5), defined as 1. At each time point, mRNA levels from REAC-treated ADhMSCs were significantly different from those detected in control untreated cells. mRNA levels from cells that had been treated with REAC in the presence of 4-MU were significantly different from the expression levels detected in REAC-treated cells in the absence of the HAS2 inhibitor. (mean ± S.E.; n = 6; P < 0.05).

**Figure 4:**
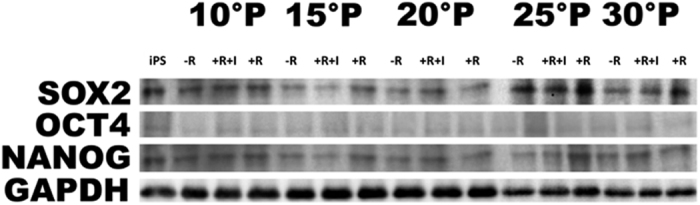
REAC-mediated rescue of Oct4 and Sox2 and NANOG protein expression is antagonized by HAS2 inhibitor. Total cellular extracts were obtained from ADhMSCs that had been exposed for 12 hours in the absence (−R) or presence of REAC (+R), or from cells that had been subjected to a 12-hour REAC treatment in the presence of 1 mM 4-MU (+R+I). Total lysate from human iPS was used as a control (iPS). Western blot analyses were performed by the aid of polyclonal antibody directed against the indicated target proteins. Representative of six separate experiments.

Figure 2 shows that increasing ADhMSCs permanence in culture up to the 30^th^ passage was associated with a progressive decline in the transcription of Bmi1, which affords chromatin remodeling to prevent cell senescence in a telomerase-independent fashion^21^,^22^,^23^,^24^. Prolonged stem cell expansion also led to a consistent decrease in the gene expression of other telomerase-independent repressors of senescence, including the stemness related genes Oct4, Sox2, and Nanog (Fig. 3). These transcriptional profiles were mirrored at the protein expression level, as it was revealed by Western blot analysis (Fig. 4).

**Figure 5:**
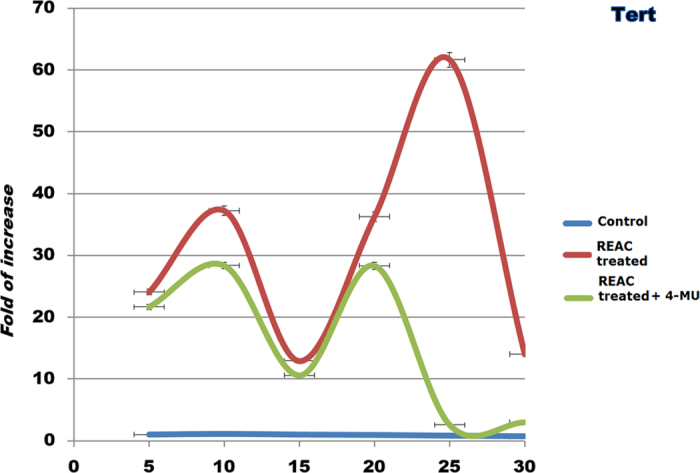
HAS2 inhibitor suppresses REAC-mediated recovery of TERT gene expression. At each indicated passage, ADhMSCs were left untreated (control) or subjected to REAC treatment for 12 hours, in the absence or presence of 1 mM 4-MU. TERT mRNA level was normalized to HPRT1 and was expressed as fold change relative to mRNA level at time 0 (unexposed cells at passage 5), defined as 1. At each time point, mRNA levels from REAC-treated ADhMSCs were significantly different from those detected in control untreated cells. mRNA levels from cells that had been treated with REAC in the presence of 4-MU were significantly different from the expression levels detected in REAC-treated cells in the absence of the HAS2 inhibitor. (mean ± S.E.; n = 6; P < 0.05).

**Figure 6:**
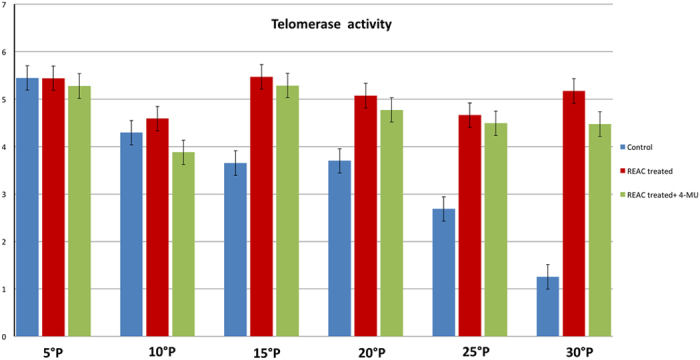
Telomerase activity. TRAPEZE-RT assay was performed in control untreated cells or ADhMSCs that had been treated with REAC for 12 h at the indicated passages in the absence or presence of 1 mM 4-MU. All data from REAC-treated cells at each time point were significantly different from those in control untreated cells. Telomerase activity from cells that had been REAC treated in the presence of 4-MU were significantly different from the activity detected in REAC-treated cells in the absence of the HAS2 inhibitor. (mean ± S.E.; n = 6; P < 0.05).

Prolonged persistence in culture also ensued into a decreased gene expression of Telomerase Reverse Transcriptase (TERT), coding for the catalytic subunit of telomerase (Fig. 5). Accordingly, telomerase activity was also inhibited (Fig. 6). Consistent with our previous findings, ADhMSC REAC treatment for 12 h counteracted the observed decline in the expression of all the investigated genes and proteins, and rescued telomerase activity, particularly at late passages (20^th^–30^th^) (Figs 1, 2, 3, 4, 5, 6).

All the antisenescence effects elicited by REAC were remarkably inhibited in the presence of the HAS2 inhibitor 4-MU (Figs 1, 2, 3, 4, 5, 6).

## Discussion

The present observations indicate that the anti-senescence action of REAC may be largely attributable to the modulation of intracellular HA homeostasis. HAS2 plays a pivotal role in preventing cell senescence, acting as a downstream target at which multiple signaling pathways converge to afford growth factor-mediated maintenance of cell differentiation^11^,^12^,^13^,^14^. Overexpression of HAS2 has also been shown to rescue the differentiating potential in aged fibroblasts, through the increased production of HA^12^,^14^. The finding that REAC action was mediated by HAS2 can also account for the ability of this technology to promote a number of crucial developmental decisions that are severely hampered in senescent cells, including the commitment to myocardial and endothelial lineages^25^,^26^. In fact, HA has been used as a component to elicit a high-throughput of cardiac differentiation in mouse embryonic stem (ES) cells^27^,^28^, to induce cardiogenesis in hMSCs isolated from different sources, including the bone marrow, the dental pulp, term placenta^27^,^29^, and amniotic fluid^30^, or to afford myocardial repair without stem cell transplantation^31^. Akin to these findings, HAS2 suppression abolished the capability of human ES cells to differentiate *in vitro* along the cardiogenic and vasculogenic lineages^10^, two lineages that represent a major developmental outcome of REAC treatment in mouse ES and human adult stem cells^25^,^26^. *In vivo*, HAS2 knockout in mice was detrimental to embryo survival and growth because of lethal cardiovascular anomalies^6^. Intracelluarly, HA exploits its multifaceted roles by acting as a docking place for hyaluronan binding proteins referred to as hyaladherins, which encompass relevant protein kinases and tissue-restricted transcription factors^6^,^32^,^33^,^34^,^35^,^36^,^37^. Most of these interactions involve molecular motors and are deployed at the level of cytoskeletal and musculoskeletal elements which form a major dynamic environment to establish and preserve cell polarity^2^. There is now increasing evidence linking altered stem cell polarity and stem cell aging^2^. In Drosophila, aged germ line stem cells showed misoriented centrosomes leading to altered polarity with respect to their stem cell niche, and reduced self-renewal activity^2^,^38^. Targeted mutation of the tumor suppressor p53 enhances symmetric division in mammary stem cells raising the chance for breast cancer development, indicating the close association between disruption of stem cell polarity and oncogenic drift^39^.

Overall, the attainment of suitable cell polarity is emerging as a universal attribute for healthy life. As a result, strategies affording a fine modulation of (stem) cell polarity may disclose unprecedented perspectives to preserve a delicate intracellular nanotopography and revert substantial traits of stem cell aging and disease. This study shows that REAC technology and hyaluron synthase 2 form an interesting network to slow down stem cell senescence. We are currently investigating whether the REAC technology may be exploited as a novel tool to act on an intracellular niche level of stem cell regulation.

## Methods

### Ethics statement

All methods were carried out in “accordance” with the relevant guidelines, including any relevant details, according to the policy approved by the local ethical committee of the University of Bologna (Title of approved project: Assessment of mesenchymal stem cells in human adipose tissue, code number 013/2010/O/Tess, date of approval from Ethical Committee: February 16th, 2010). All experimental protocols were approved by the local ethical committee. Informed consent was obtained from all subjects.

### Description of Radio Electric Asymmetric Conveyer (REAC) Technology

Radio Electric Asymmetric Conveyer Technology for therapeutic use (REAC) is a recent technology for bio and neuro modulation. The purpose of REAC technology is to optimize the ion fluxes at the molecular level, and to concentrate all the micro currents produced by these ion fluxes through the asymmetric conveyer-probe (ACP), in order to optimize the molecular mechanisms driving cellular asymmetry and polarization. The radio frequencies interact with all the structures that contain electrical charges, such as the human body, and induce currents in them. These currents vary according to the molecular characteristics of the tissues. The REAC technology generates a radiofrequency emission of very low intensity. The peculiarity of REAC technology is not the emission itself, but the particular physic link between the device and the patient’s body. The “Asymmetric Conveyer Probe” (ACP) represents this link. This is the innovation of REAC technology^40^,^41^.

REAC is an asymmetric technology, because a normal electric circuit has two physical poles: one positive and one negative (symmetrical circuit); in the REAC technology, there is only one single physical pole (asymmetrical circuit). This pole becomes the attractor (Asymmetric Conveyer) for the currents induced in the body by the radio frequency emission. This scheme has been developed for a specific purpose: to create an asymmetric circuit for better interact with the asymmetric mechanism underlying the cell polarity^42^, in order to optimize its functions. In fact, REAC technology is able to modulate the current flows existing both at cellular and body level, when these are altered. Another peculiarity of REAC Technology is the low power level used in radio frequency emission. This is necessary to induce current flows of intensity comparable with those of cell polarity. Higher power levels would disturb the adjustment mechanisms of cell polarity. Dysregulation of cell polarity can cause developmental disorders. The REAC technology is independent of the radio frequency emission used. REAC devices use only two frequencies (2.4 and 5.8 GHz). These two frequencies were chosen for two reasons. First of all, these are the most widely used and permitted at the international level. Secondly, based on our clinical and scientific experience, the 2.4 GHz frequency was chosen to better interact with tissues and cell cultures^17^,^25^,^26^,^43^,^44^,^45^,^46^,^47^,^48^,^49^,^50^,^51^, while the 5.8 GHz frequency was chosen to better interact with the nervous system. When functional modulation effects are needed, we use short duration treatments; when we want to induce developmental changes, this process of induction has to be accompanied every step of the way throughout its development. Therefore, long duration treatments are required. The REAC treatment protocol used in this study was tissue optimization (TO) regenerative treatments (RGN). The REAC model device used in this study was B.E.N.E (ASMED, Florence, Italy).

### Stem cell isolation and culture

The experimental conditions for ADhMSCs isolation and expansion *in vitro* for multiple passages are described in detail elsewhere^17^. Briefly, ADhMSCs were cultured in the presence of α-MEM, supplemented with 20% heat-inactivated FBS, antibiotics (200 units/ml penicillin, 100 μg/ml streptomycin), L-Glutamine (1%), and incubated at 37 °C in a humidified atmosphere with 5% CO_2_. Medium was changed every 4 days, and non-adherent cells were removed only after two weeks. At confluence, cells were detached with trypsin-EDTA (Sigma-Aldrich), and characterized by flow cytometry analysis and subcultured. The REAC apparatus was positioned into the CO_2_ incubator, and its ACP was immersed for 12 hours into the culture medium at each indicated passage, in the absence or presence of 1 mM 4-MU (Sigma-Aldrich), a powerful HAS2 inhibitor.

### Gene expression

Total RNA, was extracted by the Trizol reagent (Invitrogen). cDNA synthesis was performed in a 50-μl mixture containing 1 μg of total RNA and MuMLV reverse transcriptase (RT), according with the manufacturer (Invitrogen). Quantitative real-time PCR was performed using an iCycler Thermal Cycler (Bio-Rad). Two μl cDNA were amplified in 50-μl reactions using Platinum Supermix UDG (Invitrogen), 200 nM each primer, 10 nM fluorescein (BioRad), and Sybr Green. Initial denaturation step was at 94 °C for 10 min. Each cycle consisted of 94 °C for 15 s, 55–59 °C for 30 s and 60 °C for 30 s, the fluorescence being read at the end of this step. Melting curve analyses were executed to assess the product quality from RT PCR. The “delta-CT method” was used to determine the relative expression of each gene, with hypoxanthine phosphoribosyl transferase 1 (HPRT1) being considered as a reference gene. The mRNA levels from control untreated and REAC-treated cells were expressed as fold of change (2 -∆∆Ct), relative to the mRNA levels observed at passage 5 when ADhMSCs reached 80% confluence before starting REAC treatment (time 0). All primers used in this study were from Invitrogen and have been previously described^17^.

### Western blot analysis

At each indicated passage, ADhMSCs (3.3 × 10^3^ cells per well) were subjected to a 12-hour treatment in the absence (-R) or presence of REAC (+R), or they were treated with REAC in the presence of the HAS2 inhibitor 4-MU (+R+I). Induced pluripotent stem cells (iPS) from human fibroblast line (HFF1) served as positive control.

Total cell extracts were subjected to electrophoresis onto 10% Novex Tris-glycine polyacrylamide gels (Invitrogen, CA), in MOPS SDS Running Buffer. After protein transfer to nitrocellulose membranes (Life Technologies), membrane saturation and washing, a 1-h immunoreaction was performed at room temperature in the presence of the primary antibody, antisera against Oct4 (Cell signaling, Rabbit, diluted 1:200), Sox2 (Sigma diluted, Mouse, 1:200), NANOG (Cell Signaling, Rabbit, diluted 1:200) and GAPDH (Santa Cruz, Rabbit, diluted 1:200). After additional washing, membranes were incubated with anti-rabbit or anti-mouse horseradish peroxidase (HRP) conjugated secondary antibody (PIERCE, diluted 1:1000). The expression of the protein of interest was determined by a chemioluminescence method, detection system (Amersham Biosciences).

### Assessment of telomerase activity

Telomerase activity was investigated by the aid of TRAPEZE-RT (Millipore, Bedford, MA). This assay quantifies telomerase activity by measuring real-time fluorescence emission with quantitative PCR. Briefly, cells were lysed in 200 μl of CHAPS buffer. Aliquots of cell lysate (1 μg of protein/well) were assayed in a 96-well quantitative PCR plate. Wells were set aside for generation of the standard curve (TSR8 control template), negative control (no sample), and a PCR amplification efficiency control (TSK, K1). Telomerase activity (total product generated) was calculated by comparing the average Ct values from the sample wells against the standard curve generated by the TSR8 control template^17^,^52^. Assays were carried out with a CFX-96 quantitative PCR apparatus (Bio-Rad).

### SA-β-Gal staining

The “Senescence-associated β-Galactosidase Staining Kit” (Cell Signaling) was used to reveal SA-β-Gal staining. Briefly, ADhMSCs (3 × 10^3^ cells per well) were grown for 12 hours at each indicated passage in the absence of REAC, or they were subjected to a 12-hour REAC treatment in the absence or presence of 4-MU. Then, cells were fixed and processed according to the manufacturer’s instructions. For qualitative detection of SA-β-Gal activity cells were photographed (100X magnification) under an inverted microscope. The number of positively (blue) and negatively stained cells was counted in five random fields under the microscope (at 200X magnification and bright field illumination). The percentage of SA-β−Gal-positive cells was calculated as the number of positive cells divided by the total number of counted cells.

### Data analysis

Statistical analysis was performed by using the IBM- SPSS Statistics, version 22. Non-parametric Friedman and Wilcoxon Signed Rank tests were used to investigate, respectively, differences in treatments across multiple test attempts, and to evaluate, in the same group, the differences (Delta CT) between the data collected over an observational period correlated with treated or non-treated cells. A P value less than 0.05 has been considered as statistically significant.

## Additional Information

**How to cite this article**: Maioli, M. *et al*. REAC technology and hyaluron synthase 2, an interesting network to slow down stem cell senescence. *Sci. Rep.* **6**, 28682; doi: 10.1038/srep28682 (2016).
